# Heart Dose and Outcomes in Radiation Treatment for Esophageal Cancer

**DOI:** 10.7759/cureus.2378

**Published:** 2018-03-27

**Authors:** Meghan W Macomber, Stephen R Bowen, Olga Gopan, Rosanna Yeung, Smith Apisarnthanarax, Jing Zeng, Shilpen Patel

**Affiliations:** 1 Radiation Oncology, University of Washington Medical Center, Seattle, USA; 2 Radiation Oncology, University of Washington/Seattle Cancer Care Alliance Proton Therapy Center, Seattle, USA; 3 Radiation Oncology, University of Washington Medical Center, Seattle, Select Country; 4 Department of Radiation Oncology, University of Washington Medical Center, Seattle, USA

**Keywords:** esophageal cancer, radiation therapy, heart dose, trimodality therapy

## Abstract

Purpose

Studies have shown that radiation dose to the heart may be associated with worse outcomes in patients receiving chemoradiation for lung cancer. As esophageal cancer radiation treatment can result in relatively high cardiac doses, we evaluated a single-institution database of patients treated for esophageal cancer for heart dose and outcomes.

Methods

We retrospectively reviewed 59 patients with stage IIA-IIIB esophageal cancer treated with neoadjuvant chemoradiation to 50.4 Gy followed by esophagectomy from 2007-2015. Patient demographics and outcome data, including pathological response, local recurrence, distant metastases, and overall survival, were obtained. Mean heart dose (MHD), heart V5, V40, and V50, were calculated. Differences in patient characteristics between the three radiation therapy modalities: three-dimensional (3D) conformal radiotherapy (3D-CRT), intensity modulated radiotherapy (IMRT), and proton beam radiation therapy (PBT) were tested using non-parametric Kruskal-Wallis (K-W) analysis of variance (ANOVA). Patient characteristics and heart dosimetric parameters were screened by univariate Cox regression for an association to overall survival, and univariate predictors (p < 0.05) were then selected as inputs into a multivariate Cox regression model using stepwise backward elimination. Kaplan-Meier risk-stratified survival curves were plotted for the best univariate or multivariate Cox model variables. An exploratory subgroup univariate Cox regression was conducted in each of the treatment modalities (proton, IMRT, 3D-CRT).

Results

The median follow-up was 20 months. The median overall survival was 73 months. Eleven patients (20%) experienced a complete pathologic response (pCR). Only two patients (4%) experienced a local recurrence. On univariate analysis, predictors of survival were age, prior radiation, pathologic response in involved lymph nodes, and tumor length post-treatment. On a multivariate analysis, only pathologic nodal response (yN) remained significant (p = 0.007). There was no relationship between any heart dosimetric variables analyzed and any clinical outcomes.

Conclusions

In this retrospective review, radiation dose to the heart was not associated with inferior treatment outcomes in patients receiving trimodality therapy for esophageal cancer.

## Introduction

One standard-of-care option in the treatment of locally advanced esophageal cancer is neoadjuvant concurrent chemoradiation followed by esophagectomy [1]. This treatment approach has led to an improvement in outcomes over esophagectomy alone, with a population of patients expected to experience long-term overall survival. This treatment combination, however, can be associated with serious radiation-related morbidity due to the proximity of normal structures to the target volume, including heart and lungs.

Cardiac toxicity after radiation therapy has been reported in a variety of disease sites, such as breast cancer [2-3], Hodgkin’s lymphoma [4], and lung cancer [5]. Radiation-induced heart disease, including pericarditis, coronary artery disease, and heart failure, are concerns for long-term adverse effects from treatment. A study evaluating the SEER database found an increase in heart disease-related deaths in patients who received radiation therapy for esophageal cancer versus those that did not, with the risk significant at eight months [6].

Recently, it was reported in Radiation Therapy Oncology Group (RTOG) 0617, a randomized trial evaluating standard vs. high dose radiotherapy with concurrent chemotherapy in stage III lung cancer, that increasing the heart V5 and V30 significantly increased the risk of death [7]. Modern radiation therapy techniques may allow for a reduction in heart dose. The standard radiation approach for radiotherapy treatment for esophageal cancer has historically utilized three-dimensional conformal radiation (3D-CRT). Over time, treatment advances have been developed in order to attempt dose reduction to nearby critical structures. More conformal treatment techniques, such as intensity modulated radiation therapy (IMRT) and proton beam radiation therapy (PBT) have been evaluated and shown to have a similar pathologic complete response, tumor control, and overall survival rates [8-9].

While these approaches appear to be feasible and safe, it is unclear how these different treatment modalities and the different dosimetric variables affect the overall patient outcome. In this analysis, we evaluated consecutive patients who completed definitive trimodality therapy for esophageal cancer using the 3D-CRT, IMRT, or PBT approaches to assess whether heart dose negatively impacts patient outcomes.

## Materials and methods

Patients

On an IRB approved study, we identified 59 patients with stage IIA-IIIB esophageal cancer treated with neoadjuvant chemoradiation followed by esophagectomy from 2007-2015 at our institution. All patients had radiation plans that could be analyzed for cardiac dosimetry. Four patients were excluded due to inadequate follow-up data (less than one year), leaving 55 patients for analysis. Patient characteristics and outcome data were obtained via a chart review.

Radiotherapy

All patients received 50.4 Gy with external beam radiation therapy using 1.8 Gy daily fractions. The treatment approach included 3D conformal radiotherapy (3D-CRT), IMRT, or PBT at the discretion of the treating physician. Photon-based planning was performed using the Pinnacle (Philips Medical Systems, Madison, WI, US) treatment planning system. PBT was available after 2013 and delivered using the Proteus Plus System (Ion Beam Applications, Louvain-1a-Neuve, Belgium) with a mix of uniform scanning and the pencil beam scanning technique. A constant relative biological effectiveness (RBE) factor of 1.1 was used to convert physical dose to RBE-adjusted dose.

The gross tumor volume (GTV) was defined by CT findings, endoscopy report, and fluorodeoxyglucose-positron emission tomography (FDG-PET) scans when available and consisted of the primary esophageal tumor plus pathologic nodes. The clinical target volume (CTV) included a 3-5 cm craniocaudal and 1-2 cm radial expansion, and an additional 0.5-1 cm was added to the CTV to create the planning target volume (PTV).

The heart contour was redrawn by a single provider utilizing the RTOG 1106 contouring atlas. Mean heart dose (MHD), heart V5, V40, and V50, were selected for evaluation based on prior studies showing these constraints as predictors of toxicity [7,10] and were recalculated for all plans.

Chemotherapy

Chemotherapy regimens varied and were prescribed at the discretion of the medical oncologist, and thus were not included in the multivariate analysis. The most common regimen was carboplatin and paclitaxel per the chemoradiotherapy for oesophageal cancer followed by surgery study (CROSS) trial regimen [1]. Other regimens included cisplatin and 5-fluorouracil (5-FU), cisplatin and capecitabine, capecitabine and oxaliplatin, oxaliplatin and capecitabine, neoadjuvant and concurrent capecitabine, and neoadjuvant docetaxel, carboplatin, capecitabine, and concurrent capecitabine. Only four patients received adjuvant chemotherapy at the completion of treatment.

Follow-up

Local-regional recurrence was defined as a recurrence in the anastomosis or immediately adjacent lymph nodes and was confirmed with a biopsy. Distant metastases were defined as any radiographic or biopsy-proven evidence of distant disease. Patients were followed at provider discretion, but generally were seen within three months of treatment, and then every three to six months for two years and annually until five years. All patients included had at least one year of follow-up.

Statistical analysis

Differences in patient characteristics between the three radiation therapy modalities (3D-CRT, IMRT, and PBT) were tested using non-parametric Kruskal-Wallis (K-W) ANOVA. Patient demographics and heart dosimetric parameters were screened by univariate Cox regression for an association with overall survival. Heart dosimetric parameters were tested as both continuous variables and as dichotomized variables with cutoffs defined by the Youden index of accuracy (sensitivity + specificity – 1) under a receiver-operator characteristic (ROC) analysis. Univariate predictors (p < 0.05) were then selected as inputs into a multivariate Cox regression model using stepwise backward elimination. The final Cox model maximized log-likelihood and minimized cross-correlation between predictors. Kaplan-Meier risk-stratified survival curves were plotted for the best univariate or multivariate Cox model variables. An exploratory subgroup univariate Cox regression was conducted in each of the treatment modalities (PBT, IMRT, and 3D-CRT).

## Results

Patient characteristics are listed in Table 1.

**Table 1:** Patient characteristics All values represent n unless otherwise specified; continuous variables are reported as median. 3D CRT: 3D conformal radiation therapy. IMRT: Intensity-modulated radiation therapy. PBT: Proton beam radiation therapy. GEJ: Gastroesophageal junction. pCR: pathologic complete response. U: endoscopic T and N stage pre-treatment. Y: pathologic T and N stage. K-W: Kruskal-Wallis. COPD: Chronic obstructive pulmonary disease.

	3D-CRT	IMRT	PBT	K-W p
Age	67.5	61	62	0.07
Gender (%male)	61	71	88	0.83
Smoking history				0.2
Never	5	9	7	
History	11	9	8	
Current	0	3	1	
Comorbid disease				
Hypertension	7	11	12	0.4
Hyperlipidemia	6	6	12	0.09
Cardiac arrhythmia	0	2	1	0.46
Coronary artery disease	1	5	4	0.34
Diabetes mellitus	0	5	4	0.11
COPD	2	1	1	0.64
Prior radiation	1	2	1	0.88
uT		1 unknown		0.62
uT2	2	4	3	
uT3-4	16	16	15	
uN				0.52
uN0	1	4	6	
uN1-2	17	17	10	
Stage				0.18
IIA	0	1	2	
IIB	3	7	6	
IIIA	12	11	7	
IIIB	0	2	3	
IIIC	1	0	0	
pCR	4	4	3	0.83
yT				0.72
yT0	4	4	3	
yT1-3	12	17	15	
yN				0.08
yN0	10	9	12	
yN1-2	6	12	6	
Tumor location				0.69
Mid	3	3	4	
Distal/GEJ	13	18	14	
Tumor length- pre	5.5 cm	4 cm	5 cm	0.01
Tumor length- post	0.1 cm	0.9 cm	0.9 cm	0.89
Histology				0.04
Adenocarcinoma	10	18	17	
Squamous cell	6	3	1	
Chemotherapy				0.13
Carboplatin/paclitaxel	10	19	18	
5-Fluorouricil/cisplatin	1	1	0	
Capecitabine/oxaliplatin	2	1	0	
Other	3	0	0	
Adjuvant chemotherapy	0	4	0	0.12

Pre-treatment staging ranged from stage IB-stage IV, with the majority stage IIB; all patients were treated with curative intent. There were 18 patients treated with 3D-CRT, 21 patients treated with IMRT, and 16 patients treated with PBT. The median age was 62 years. The majority were male (73%) with adenocarcinoma histology (82%) located in the distal esophagus/gastroesophageal junction (82%). Among baseline clinical characteristics, the only differences across the treatment modality subgroups were pre-treatment tumor length (longer median length in patients undergoing 3D CRT, p<0.01) and histology (more patients undergoing 3D CRT with squamous cell carcinoma, p < 0.04).

MHD was 3090 cGy (range 269-4757 cGy). The highest heart dose was in patients treated with 3D CRT (34.8 Gy) and was significantly lower in patients treated with PBT (9.7 Gy), as would be expected with the different dosimetric properties of these treatment modalities (Table 2).

**Table 2:** Dosimetric data Dosimetric data for the heart shows that 3D-CRT had the highest values for all parameters evaluated, and PBT had significantly lower values.

	3D-CRT	IMRT	PBT	K-W p
Heart mean dose	34.8 Gy	25.8 Gy	9.7 Gy	< 10^-8^
Heart V5	96%	98%	32%	< 10^-6^
Heart V40	39%	18%	12%	< 10^-4^
Heart V50	10%	5%	4%	0.06

However, these differences did not correlate with any difference in treatment outcomes. There was no correlation between stage, location or tumor length with heart dose.

Median follow-up was 598 days (20 months). Median overall survival (OS) was 73 months, with one-year OS 92% and two-year OS 77%. Eleven patients (20%) experienced a complete pathologic response (pCR). Only two patients experienced a local recurrence. On univariate analysis, heart dose was not a predictor of local recurrence, distant metastases, or overall survival. Screened predictors of survival include age, prior radiation, pathologic response in involved lymph nodes, and tumor length post-treatment (Table 3).

**Table 3:** Significant results of univariate analysis of patient characteristics (Table 1) and overall survival

	Hazard Ratio (95% CI)	p-value
Age	1.1 (1.0-1.2)	0.008
Prior RT	3.9 (1.2-12.3)	0.02
yN	2.1 (1.3-3.3)	0.003
yN+ (yN1-3 vs yN0)	24.7 (3.2-187.5)	0.002
Tumor length post-treatment	1.3 (1.0-1.7)	0.05

In a subgroup univariate analysis by treatment modality, in the 3D CRT group, yN remained significant (16 patients, six events), heart rate (HR) = 3.1, p = 0.04. In the IMRT group, yN remained significant (21 patients, six events), HR – 2.9, P = 0.03. In the proton group, age remained significant (18 patients, four events), HR 1.2, p = 0.03.

On multivariate analysis, only pathologic nodal response (yN) remained significant for OS (p = 0.007) (Table 4) following stepwise backward elimination to maximize log-likelihood (p = 0.006) and minimize variable cross-correlation (R < 0.007).

**Table 4:** Multivariate analysis Remaining significant variables after a multivariate analysis of patient characteristics (Table 1) and overall survival.

	Hazard Ratio (95% CI)	p-value
yN	2.0 (1.2-3.5)	0.007
Tumor length post treatment	1.3 (1.0-1.7)	0.10

**Figure 1:**
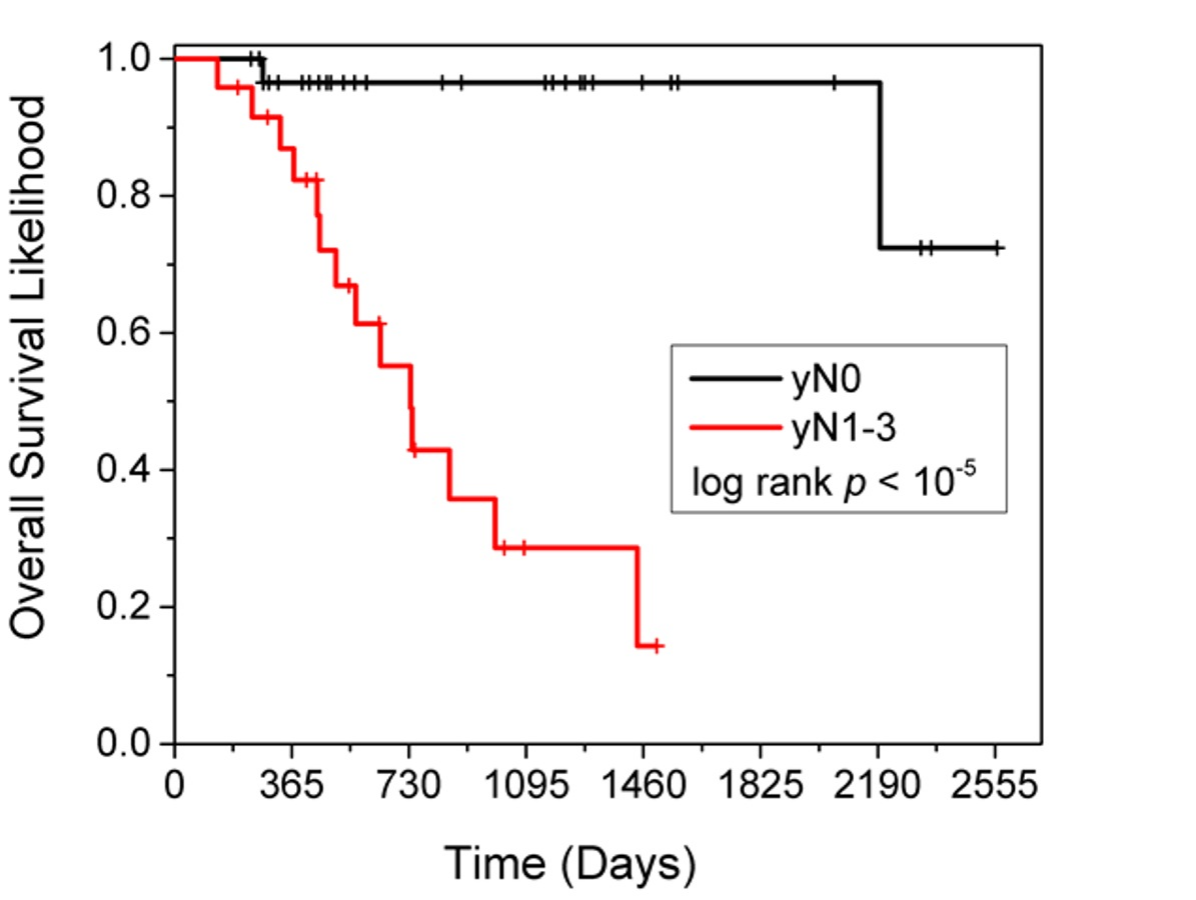
Risk-stratified KM curves showing the best and univariate and only multivariate predictor of overall survival: pathologic nodal response KM: Kaplan-Meier

Figure 1 shows risk-stratified KM curves based on the best univariate and only multivariate predictor of OS (yN status). There were no significant interactions for heart dose with treatment modality, any clinical variable, or any dosimetric variable analyzed.

## Discussion

In this analysis of patients treated with definitive trimodality therapy for esophageal cancer, with a 20 months' median follow-up, the mean heart dose, V5, V40, and V50, were not predictive of local recurrence, distant metastases, or overall survival. We did not see any difference in pathologic complete response, local or distant recurrence, or overall survival among patients treated with 3D CRT, IMRT, or PBT. Although we did not see any correlation between heart doses and survival, we did find a strongly statistically significant increase in overall survival for patients who had no evidence of lymph node disease after treatment compared with those that did (yN0 vs yN1-3), p < 0.007.

Data on cardiac mortality after radiotherapy for esophageal cancer is limited, perhaps because of the previously low cure rates after treatment. However, with contemporary treatments, there are increasing long-term survivors of this disease. In recent years, several reports have been published detailing cardiac toxicity after radiation treatment, such as in breast cancer, Hodgkin's lymphoma, and lung cancer [2-5]. Esophageal cancer treatments involve significantly higher doses to the heart, and it is unclear what impact this may have on patient outcome. It was recently reported in RTOG 0617 that the heart V5 and V30 are important predictors of patient survival [7]. However, older studies in non-small cell lung cancer (NSCLC) found no correlation between heart dose and cardiac events or survival. It has been suggested that the location and extent of mediastinal lymph node involvement correlate with heart dose and, thus, may be a surrogate for more advanced disease [14].

Other possible explanations for our findings include the fact that patients may have been too young and/or the follow-up is too short for heart dose to translate into an adverse clinical outcome. A review of current literature reporting on the types and incidence of cardiac toxicity after multimodality therapy found that most events occurred within two years of treatment and that older age and female sex posed a higher risk [15].

It has been shown in numerous studies that a pathologic complete tumor response is associated with improved survival outcomes [1,10]. Furthermore, numerous prior studies have found that residual nodal disease after CRT is the most important prognostic factor [11-13]. This implies that response to treatment is more important than treatment-related toxicity in regard to patient outcome, and for this reason, the focus of future treatment modalities should be on aggressive therapies in this disease with currently poor overall outcomes to treatment.

The standard radiation approach for radiotherapy treatment for esophageal cancer has historically utilized three-dimensional conformal radiation (3D-CRT). Over time, treatment advances have been developed to attempt dose reduction to nearby critical structures. More conformal treatment techniques, such as IMRT and PBT, have shown a similar pathologic complete response, tumor control, and overall survival rates [8-9]. We did not find any differences in outcome in the different treatment modalities, although there was a significant decrease in heart doses with PBT. As this analysis did not evaluate specific cardiac toxicity endpoints, future investigation on the impact of PBT and a reduction in clinical cardiac toxicity is warranted.

There are several limitations to this study. There may be an inherent bias in patient selection due to the retrospective nature of this analysis and the heterogeneous patient characteristics and treatments. Patients may have been selected for different treatment techniques for a variety of reasons; for example, patients with underlying comorbidities, such as underlying cardiac disease or a history of prior radiotherapy, may be more likely to undergo PBT, although our patient groups were well-balanced between the IMRT and PBT arms. It is a relatively small number of patients, which has limitations for statistical analysis. Additionally, we were not able to address specific cardiac events. Future prospective studies evaluating treatment outcomes in esophageal cancer should address heart dose as an independent predictor of outcome.

## Conclusions

In conclusion, a radiation dose to the heart was not associated with inferior treatment outcomes. However, overall survival was significantly improved in patients who achieved a complete pathologic nodal response to neoadjuvant chemoradiation therapy. Clinical strategies for improving outcomes in esophageal cancer should involve innovative ways to enhance the multimodality treatment regimen in order to achieve a pathologic complete nodal response.
